# The evolution and adaptation of evidence synthesis during the COVID-19 pandemic in Canada: Perspectives of evidence synthesis producers

**DOI:** 10.1371/journal.pone.0314657

**Published:** 2024-11-27

**Authors:** Tricia Corrin, Eric B. Kennedy

**Affiliations:** Disaster and Emergency Management, York University, Toronto, Ontario, Canada; Western University Faculty of Science, CANADA

## Abstract

The demand for evidence syntheses to inform urgent decision-making surged during the pandemic. The challenging circumstances of the pandemic created significant hurdles for both those requesting and creating evidence syntheses, leading to the refinement and adjustment of evidence synthesis practices. This research sought to capture and explore how the field of evidence synthesis evolved and adapted during the pandemic from the perspective of those who produced evidence syntheses in Canada. In this qualitative study, semi-structured interviews were carried out between October 2022 to January 2023. Twenty-two participants from 19 different organizations across seven provinces and one territory were interviewed. This included producers of evidence syntheses from academic institutions, not-for-profit organizations, and provincial and federal government. Data analysis was conducted thematically using a phenomenological approach. Results indicated the evidence synthesis landscape drastically changed during the pandemic including short timelines to produce syntheses and changes in the volume, types, and quality of literature included in them. Due to the changing landscape and different needs of requestors, evidence synthesis methodologies evolved, synthesis products were tailored, and quality assessment tools were adapted. In addition, the use of artificial intelligence, processes for engaging subject matter experts and patient-citizen partners, and the coordination of the evidence synthesis community changed. The findings of this study contribute to the ongoing dialogue surrounding evidence synthesis to inform decision-making, and highlights the importance of flexibility and necessity of continuously evolving methodologies to meet the demands of frequently changing landscapes. The lessons learned from this study can help inform future strategies for improving evidence synthesis practices not only in the face of public health emergencies, but also in everyday practice.

## Introduction

The COVID-19 pandemic created an urgent demand for evidence to understand the virus and formulate effective public health responses. Initial information on the virus was limited due to its novelty, but this quickly changed with the rapid generation of information from different sources around the world [1, 2]. The deluge of scientific literature, coupled with the rapidly evolving pandemic, posed significant challenges for researchers and decision-makers to keep up-to-date of the most accurate evidence [3–7].

Evidence synthesis is a research methodology that involves consolidating information from all relevant evidence on a topic to comprehensively understand the findings [8]. These syntheses assess all the studies on a topic together in context to provide a comprehensive and transparent basis for applying research to decision-making [8]. Although it has a long history in the field of health sciences, evidence synthesis is now used across multiple disciples, and is considered by many as an essential part of assessing evidence on societally relevant questions to inform policy [9]. Systematic reviews, often considered the “gold standard” by some supporters of the evidence-based medicine movement, occupy the top tier of the “evidence hierarchy,” a classification system used to inform decision-making and underpin evidence-based practice [10, 11]. Over time, various types of evidence syntheses have emerged to accommodate diverse research questions, meet the requirements of decision-makers, and address time constraints including rapid reviews, scoping reviews, realist reviews, and living reviews, among others [12].

While systematic reviews are highly valued inputs to evidence-based decision-making, their rigorous and time-consuming methodology, including assessing evidence quality and certainty, can present challenges [13, 14]. Systematic reviews are not always possible or ideal depending on factors such as the research question, type of evidence underpinning the research question, context, or time constraints [14, 15]. Evidence syntheses are also not immune to political controversy, such as criticisms of being manipulated to achieve particular political outcomes, such as the Cochrane review on the effectiveness of face masks in reducing the spread of COVID-19 [16, 17].

In Canada, evidence-informed decision-making is a key feature of the public health system and has government endorsement [18, 19]. The government’s public messaging emphasized that the approach to managing the COVID-19 pandemic and creating related policies was grounded in scientific evidence [20, 21]. However, the pandemic created challenging circumstances for the generation of evidence syntheses to aid in evidence-based decision making. With respect to evidence found in the scientific literature, challenges included the rapid generation and changing of evidence, a lack of evidence in certain areas such as pathogenesis, treatment options, and transmission dynamics, most prominently at the beginning of the pandemic, and variations in the quality of evidence [1, 2, 22]. Numerous deficiencies within the systems responsible for generating evidence were exposed which lead to significant challenges for those trying to interpret the evidence [23]. This included inadequate coordination of research efforts, poorly conceived study designs such as clinical trials, and the duplication of research efforts [23, 24]. These challenging circumstances coupled with the demand for the rapid generation of evidence syntheses for decision-making prompted adaptations and the development of new systems and frameworks within existing evidence synthesis methodologies [1, 7, 25–27].

Research has begun to emerge on the challenges, modifications, and implications for the field of evidence synthesis during the pandemic; however, investigations into this topic are still in the early stages of development [7, 25–27]. It indicates that an unprecedented volume of evidence syntheses was both requested and generated during the pandemic, necessitating significant adaptations within the field to fulfill these demands. There is a need for more in-depth case studies on how suppliers of evidence syntheses responded. To further this understanding, a qualitative study was conducted to examine how evidence synthesis evolved and adapted during the COVID-19 pandemic in Canada, as perceived by those who were actively involved in producing them.

## Methods

### Research question

Using a phenomenological approach, this study aimed to answer the following research question:

As a result of the global COVID-19 pandemic, how did evidence synthesis evolve and adapt in Canada to produce evidence syntheses for evidence-based decision-making?

### Research approach

#### Study design

The qualitative research design utilized semi-structured interviews to delve into the experiences of individuals in the field, with the aim of capturing their perspectives on the research question. The creation of semi-structured interview questions drew upon the researchers’ pre-existing knowledge from working in the field of evidence synthesis during the pandemic (TC) and experience in critical science studies (EBK) (S1 Appendix). To ensure the clarity and coherence of the interviews, and to test for alignment between the intended questions and respondents’ interpretations, pilot interviews were conducted (TC) with three individuals working in the evidence synthesis field between September 28–30, 2022.

#### Data collection

A combination of knowledge from the researcher and a search of government and academic websites for evidence synthesis groups was used to identify and recruit participants between September 12 –November 18 2022. Eligibility for the study required individuals (aged 18+) to have worked in the field of evidence synthesis in Canada during the pandemic. For groups identified from the website search, the first participant with contact information available was selected for participation. For larger organizations that had multiple evidence synthesis groups, a participant was selected for participation from each group. If there was no response, the second person would be contacted. Until data saturation occurred, participants were recruited through email and snowball sampling to identify participants not found through the search. Data saturation was reached when no new information was emerging from the interviews [28, 29].

Only one person declined participation, redirecting to another team member. No incentives were offered for participation. Prior to interviews, each participant received an overview of the study and an informed consent form to review and sign. Information about the researchers’ background (gender: female, credentials: Master of Public Health, occupation at time of study: evidence synthesis producer, experience: no qualitative research experience, topic expert) and interest in the research topic was provided, along with an opportunity for participants to seek clarification. All interviews were conducted using Microsoft (MS) Teams by TC between October 20, 2022 and January 6, 2023 and were recorded in both audio and visual formats. Twenty-two participants spanned 19 organizations and seven provinces (Alberta, British Columbia, Manitoba, Ontario, Quebec, Newfoundland, Nova Scotia) and one territory (Yukon). The organizations included academic institutions (n = 7), non-profit organizations (n = 8), and provincial (n = 2) and federal government (n = 2). The majority of the participants were well established professionals in the field of evidence synthesis and were either the managers or team leads of their evidence synthesis groups. Five participants were acquainted with the researcher through current and previous working relationships. The interviews averaged 60 minutes, with durations ranging between 45–90 minutes. Transcripts were not returned to the participants for corrections.

Most evidence synthesis groups interviewed were well established prior to the pandemic, with only three groups initiated as a result of the pandemic. Prior to the pandemic, these evidence synthesis groups worked on a large range of topics which included but are not limited to: infectious and chronic diseases, cancer, immunization, antimicrobial resistance, preventative healthcare, infection prevention and control, health systems performance, effectiveness of pharmaceuticals and interventions, new healthcare technologies and medical devices, workplace health and safety, emergency care, pediatrics, gerontology, and social determinants of health.

#### Data analysis and reporting

The interviews were transcribed using MS Word’s built in automatic transcription, then verified manually against the audio files. Prior to analysis, a bracketing exercise was conducted to articulate the researchers’ own assumptions. Using a combined deductive and inductive approach, a codebook for interview coding was generated (S1 Appendix). A horizontal analysis on two interview transcripts was performed to identify potential codes within each individual interview followed by a vertical analysis to identify common codes across the interviews using three additional interviews [30]. The researcher then integrated their assumptions with the generated codes and interpretations to establish final codes and overarching themes. Transcribed interviews were imported into the qualitative data analysis software MAXQDA (VERBI Software, 2021) for coding and analysis. To assess inter-rater reliability, one interview was recoded twice, both at the beginning and end of the process. Using the coded data, a thematic analysis was conducted. The study’s reporting adheres to the Consolidated criteria for REporting Qualitative research (COREQ) checklist (S2 Appendix) [31].

#### Ethics

Ethical approval was obtained through York University (#E2022-277). Participants provided written informed consent. No minors were interviewed.

## Results

The evolution and adaptation of evidence synthesis during the pandemic was explored from the perspective of those who produced it in Canada. First, an overview of how the landscape changed as a result of the pandemic is presented. From there, we explore how the field of evidence synthesis evolved and adapted in a challenging environment.

### 1.0 Changing landscape

The landscape in which evidence synthesis producers conducted their work drastically changed during the pandemic:

*“So you have this perfect storm of more evidence, less time, and really poor quality and with decision makers looking for an answer […] and so there was always, you know, that combination, something you don’t really want to see in a perfect knowledge synthesis world.”* [interviewee 3]

Specific changes included timelines, literature volume, varied types of evidence, use of pre-prints, and evidence quality considerations.

#### 1.1 Timelines to produce evidence syntheses

To meet the needs of decision-makers, evidence syntheses requests featured dramatically shorter-than-usual timelines ranging from hours to weeks:

*“We went from some of our scoping reviews taking one to two years, a good systematic review six months to a year…[to] them asking us to do something in three days.”* [interviewee 7]

All participants expressed frustrations with the expedited timelines as they felt were not conducive to producing quality products:

*“It’s sometimes crazy what the level of expectations of decision makers are, how long they expect things to actually take to do properly, as compared to what it actually does take to do properly.”* [interviewee 3]

While timelines lengthened as the pandemic progressed, participants expressed that this was a common frustration throughout its duration.

#### 1.2 Keeping up with the literature

Due to the volume and the speed at which evidence was emerging, participants found it difficult to keep up with the literature and subsequently keep evidence syntheses up to date:

*“The evidence was changing on almost a daily basis. I remember us publishing one review…we had searched the evidence, updated the search three days before we published it. We published it on a Friday and someone emailed us on a Monday and said you missed these three studies that came out and it was like, that would just not happen outside of a public health emergency, where evidence on a specific topic could completely change.”* [interviewee 5]

Many expressed that by the time they were able to complete a synthesis early in the pandemic, it would already be outdated.

#### 1.3 Searching and utilizing different type of evidence

Participants expressed that they had to adjust search methods and use different types of evidence they may not have normally considered early on when there was little to no evidence on a topic. This included using grey literature (e.g., government reports) and preprints. In many instances, late-breaking evidence sources such as preprints were found from Twitter, popular press, or communal networks and listservs:

*“I would have this kind of grapevine knowledge just from being really, really hooked in [on Twitter]…You wouldn’t have found the preprints, you wouldn’t be aware of the work being done, and so that kind of really dynamic knowledge ended up being super useful.”* [interviewee 7]

Even as peer-reviewed publications started to emerge, grey literature and preprints continued to be used as sometimes they contained more up-to-date information. The use of preprints was novel to all participants during the pandemic. With the exception of one participant, all included preprints in their evidence syntheses and did not feel like they had a choice in the matter:

*“It wasn’t a question of whether we included preprints or not, because we knew that in the COVID world, you have to include prints.”* [interviewee 12]

As the pandemic progressed, a few noted that the use of preprints was dependent on the topic and scope. Many mentioned that although it was necessary to use preprints, they posed multiple challenges and created a lot of discussions on how and when they should be used.

The main issues raised with preprints were the difficulty searching preprint servers and knowing when a preprint has been published. Initially, the preprint servers were not set up for searching using Boolean logic or exporting the results, which created a lot of manual work. In addition, as preprints were subsequently published, the information from the published version would have to be updated in the syntheses:

*“It was really hard for us to deal with from a synthesis perspective when we were updating or doing a review. The preprint server, in theory, is supposed to show when it’s actually been published but depends, some journals do it automatically, others do not. Sometimes the title would change, sometimes the author order would change from preprint to publication. The data would change altogether, and so we had to be really thorough. It was a huge extra step to make sure we were not double counting the study by not realizing that was the preprint which has now changed.”* [interviewee 5]

Many expressed frustration at this very manual and cumbersome process.

#### 1.4 Quality of evidence

There were many questions and frustrations about the quality of the evidence during the pandemic. While participants understood that types of studies regarded by many evidence synthesizers as preferable for decision making, such as randomized controlled trials, were not available at the beginning of the pandemic, many noted that there was an influx of science that was poorly conducted and reported:

*“The difficulty as well with knowledge synthesis during the pandemic is that there is a lot of less than ideal studies and study designs […] the majority of them aren’t what you’re hoping to have when it comes to doing an ideal sort of review, you really want high quality studies, but it’s difficult to get that, and so that has been the challenge.”* [interviewee 8]

Participants felt this made it difficult to find and use the best available evidence to answer important research questions and often reported the inclusion of preprints as a limitation in their syntheses. Some also began to question the peer review process:

*“An additional challenge was this issue of calling into question the entire peer review process which I don’t think has a leg to stand on anymore. It has to be at the least interrogated, you know, at the most reconceptualized and completely reimplemented because it seems like it’s in shambles at the moment.”* [interviewee 19]

### 2.0 Evolution of evidence synthesis

As a result of the changing landscape, time constraints, and different needs of requestors, participants described evidence synthesis methodologies evolving over the pandemic.

#### 2.1 Changes to evidence synthesis products

Some participants continued to produce the types of products they always had been generating, while many shifted to different products to meet the immediate demands for evidence.

At the beginning of the pandemic when information was starting to emerge, multiple evidence synthesis groups independently started producing synopses of key articles. This involved searching the literature on a daily or weekly basis and creating reports on what new evidence had emerged. Many of the groups were not aware that others were doing the exact same thing just for different requestors.

To account for some of the limitations in the evidence, time, and background knowledge of end-user, participants described adaptations that were made to traditional evidence syntheses products to improve their utility. Many created new templates and restructured the reporting of their evidence syntheses:

*“What defined the reports that we did during COVID to capture ‘what did the decision makers care about? How could we make this document as useful to them as possible?’ So we flipped the structure, instead of putting methods up front, we put them at the back […]there was the kind of conventional look through everything, do a good write up that includes some discussion, like picking out the higher quality pieces, then we took that and synthesized it down to key messages and synthesized those into recommendations in a lay summary. So a lot more synthesis pieces.”* [interviewee 18]

In addition to restructuring traditional evidence syntheses there was a need to produce much shorter products that communicated the key take home messages leading to the production of 1–3 page synthesis products.

Several participants believed it was important to include contextual information in the products so that end-users had a full understanding of the issue and everything surrounding it. This involved including things that would not traditionally be found in evidence syntheses like how the issue was portrayed by the media. One of the biggest changes noted was the inclusion of practical guidance and recommendations so that requestors would have something to work with:

*“We would have some draft recommendations so that the people receiving the report would actually have something to discuss because we found if we just kind of gave the information, it wasn’t helping them make decisions and so we would actually try to answer the questions with what we thought were some recommendations […] and a rationale because then they had kind of a straw dog to actually talk about what they might consider doing or why that may or may not be possible.” [interviewee 7]*

Since there were many different requestors and needs throughout the pandemic, there was no universal standards for these products.

*2*.*1*.*1 Rapid reviews*. As many participants were asked to generate evidence syntheses under extremely tight timelines, many shifted to producing rapid reviews or very short custom syntheses instead of systematic reviews. They noted that there continued to be no universal consensus as to what a rapid review was and which shortcuts were appropriate to take under what circumstances:

*“It’s really balancing out speed and efficacy of the process itself by taking shortcuts into the development of the process. How you make those shortcuts differs from each group and almost no two groups are the same. There will be no two groups that have come to the same conclusion on the benefits/harms or risks associated with short cuts that they take.”*[interviewee 3]

Participants described many different shortcuts to conducting rapid reviews during the pandemic including not registering their protocol, using a single reviewer (most common), searching a limited number of databases, forgoing a second review on the search strategy, using artificial intelligence (AI), and not conducting quality assessment. Other participants conducting rapid reviews stated they did not take any methodological shortcuts, but instead either narrowed the scope of the research question or increased the size of their group to complete the synthesis:

*“Rapid, it just means more people working quicker, together, it doesn’t actually mean that you didn’t do the process. That’s what happened at least in the beginning of COVID, large groups of people came together, we got just got a lot of people working very quickly to screen through studies and we were able to produce a review very rapidly.”* [interviewee 20]

While most participants recognized the increased popularity of rapid reviews was due to the nature of the pandemic, many believed they have become more widely accepted and are here to stay. However, a few expressed frustration by the rise in their acceptance:

*“Today I think it’s anyone can slap the word rapid review on it and be ‘well you know, of course I didn’t follow standard methods because it’s a rapid review’, and that would be acceptable.”* [interviewee 3]

Many were in consensus that there are still a lot of unknowns about using rapid reviews:

*“I think there is a time and place for rapid reviews, but I also feel that we aren’t able to fully communicate or fully know the potential harm of using a rapid review in some situations.”* [interviewee 14]

Overall, all participants agreed there should be more discussions around rapid reviews regarding what they should be used for, what shortcuts are appropriate in what circumstance, and the potential harms of using rapid reviews.

*2*.*1*.*2 Living evidence reviews*. As a result of the rapidly evolving evidence, participants noted the creation of living evidence reviews also gained traction during the pandemic:

*“We moved fairly quickly to an evolving suites of living evidence syntheses, holy shit, I mean that was a real game changer when that started to happen.”* [interviewee 16]

While some naturally moved to a living evidence review after being asked to update their rapid review multiple times as information became outdated, others identified from the start that this was the best method under the circumstances. Living reviews were a good way to keep track of evolving information:

*“We were trying to grab or identify all the little bits and pieces of information as quickly as possible to help keep ourselves organized. It was being done really quickly, often by one person screening, one person extracting. These things weren’t being double reviewed because there simply was not time to do so. So those initial projects, the living evidence summaries, living evidence tables maybe is a better term for it, but they were literally kind of a one stop shop for here are the 10 articles on latent period.”* [interviewee 21]

While living reviews were generally thought of positively, there were also some challenges noted to producing them such as being time and resource intensive, requiring constant adaptation, and difficultly maintaining consistency:

*“So here’s the rub, right? When you do a living review it’s always time limited, so anything you add you have to take something away because there’s only a finite amount of time and if you’ve got to turn it around in a less than 10 day window, you can’t have too many moving parts because it just it implodes upon itself. That was another interesting piece of this living review is that where it started from and where it is now, is different […]. How do you make sure you still got consistency across the rounds that you do and that what you’re pulling now is not fundamentally different from what you’ve pulled before?”* [interviewee 12]

Of those conducting living evidence reviews, they were often updated weekly initially and slowed to biweekly as they became more populated with information that wasn’t changing. Many were stopped once there was either too much literature on the topic to be feasible to continue, or the literature on the topic was no longer changing.

### 2.2 Adaptation of quality assessment tools

There was a small number of participants who conducted systematic reviews during the first year of the pandemic which included appraising the quality of the evidence (risk of bias assessment and assessing certainty of the evidence). Often it was conditional on how many studies there were and how much time they had to complete the evidence synthesis. Some did not use any standard tools to critically appraise the evidence, but instead made high level comments as to the quality and certainty of the evidence based on direction of evidence and study designs:

*“We were not quality assessing things, we would note what the study design was and for the most part that gives a pretty good indication of what the level of evidence is, but we would not be able to fully unpack that and then synthesize things together for people.”* [interviewee 21]

A few participants noted that they made adaptations to the process:

*“We would actually do modified risk of bias assessments, and then you’d say ‘well, these are all super biased, but at least the bias is in the same direction’, but it was a lot less, it would basically be top line summary type more so than the very detailed GRADE or other methods.”* [interviewee 7]

Other adaptations included using shorter and simpler risk of bias assessment tools such as the Ottawa Newcastle Scale instead of more comprehensive tools such as ROBINS-I, or modified existing tools.

#### 2.3 Use of artificial intelligence (AI)

With the large volume of literature and the speed at which evidence syntheses were being requested, participants believed AI tools were beneficial in creating efficiencies in the evidence synthesis process. However, not everyone had access or chose to utilize AI tools. AI tools found within software such as DistillerSR were used to accelerate parts of the evidence synthesis process. The most commonly noted efficiencies included using ranking features to sort articles by relevance and using it to act in place of a second reviewer for screening. Participants planned on either adopting or refining the tools in the future, especially to automate some of the tasks that do not require evidence synthesis expertise:

*“We’re going to look at more use of AI technologies, and particularly for some of the stuff that took a lot of tinkering, like with different databases and moving A to B to C […]. maybe some of that could be automated a little bit more so that our expertise could be put into more of the synthesis side of things and extracting the information rather than just identifying and looking for information.”* [interviewee 21]

#### 2.4 Subject matter experts/patient-citizen partners

As there was often not enough time for evidence syntheses to go through the peer-review process, many participants used subject matter experts on the topic to review the products before they were sent to decision-makers.

Prior to the pandemic some participants utilized and engaged patients and citizens when designing and conducting evidence synthesis to ensure their voice and priorities were being considered. However, during the pandemic, this became more standard practice for some evidence synthesis producers:

*“The other game changer with COVID evidence synthesis was moving to citizen involvement in those processes […].. This is citizen engagement in the synthesis of existing evidence on very short timelines to meet a decision makers request and it’s just been a game changer for us and so we will not go back the old system of just doing these for a policymaker audience. We are trying to figure out how do we move to a new system where we have citizen partners in all of this evidence synthesis work even when it’s done under these very tight timelines.”* [interviewee 16]

#### 2.5 Open science

Many participants expressed gratitude for journals making COVID-19 literature open access and the importance of open science during the pandemic. All participants tried to adhere to open science concepts as much as possible and many felt that it was one of their core values:

*“In the interest of academic research and transparency, if somebody wants to look at what we’ve done, it’s all there, it’s all available, it’s all online, there’s no black box, there’s no hocus pocus.”* [interviewee 19]

This included making their work publicly available on websites and open science platforms. This was a major shift from disseminating their evidence syntheses via peer-reviewed publications to sending it directly to the end-user, using an email distribution list, submitting as a preprint, positing it on social media, or adding them to a website.

While many noted that it would have been ideal to submit their evidence syntheses to a journal for publication, some believed that since their work was being done so rapidly, with so many shortcuts, that their work was not appropriate to submit. In addition, by the time an evidence synthesis made it through the peer-review process, it would likely be out of date and often no longer relevant:

*“It’s challenging publishing because, we’ve had a few papers rejected because now the search is 10 months old and in COVID, that’s not acceptable or the decisional dilemma has changed now and nobody in Canada cares about first vaccination priority anymore because everyone’s vaccinated now, so your studies no longer of interest to the public.”* [interviewee 9]

There were also some challenges to making evidence syntheses available when complete. While some participants posted their products on websites prior to the pandemic and could continue to do so, others found this process difficult due to organizational roadblocks:

*“I’m sure there’s a better way to disseminate information, so trying to jump some of those hurdles, and some of them are agency red tape, like web security tape hurdles in anticipation that this is probably not the last time that we’re going to be in potentially a response mode”* [interviewee 21]

A few noted that there was not enough open science during the pandemic which was a barrier. Most notably, participants found it difficult to find others’ search strategies and lists of excluded studies with reasons for exclusion for their evidence syntheses. Making these available would have been helpful to help other teams piggyback on the work that is already been done to save time and people power.

#### 2.6 Evidence synthesis community

Although there have always been collaborations between evidence synthesis producers, the vast number of questions that required evidence syntheses facilitated the need for more collaboration:

*“COVID was kind of a wakeup call that there were all of these little disparate evidence synthesis groups around, mostly across provinces, across institutions and that coordination work is needed.”* [interviewee 18]

Many noted that collaborations substantially increased during the pandemic, including across provinces and with international organizations. Collaborations were much easier due to technological advances such as the use of Zoom to communicate. At the beginning of the pandemic participants coordinated and collaborated with people they knew in their research community through a variety of platforms such as Twitter, WhatsApp, and email. Some of the reasons to collaborate included being asked to produce evidence syntheses on similar questions, share resources, and provide feedback (e.g., on search strategy or research question etc.)

Participants cited the COVID-19 Evidence Network to Support Decision Making (COVID-END) developed out of McMaster University and the National Collaborating Centre for Methods and Tools COVID-19 public health review repository as initiatives that were valuable in coordinating evidence synthesis efforts and minimizing duplicate efforts during the pandemic across Canada. The purpose of COVID-END was to coordinate the COVID-19 evidence syntheses being produced and maintain an inventory of COVID-19 evidence syntheses that was fully searchable. Most participants interviewed were a part of the COVID-END initiative and thought highly of it. Through this initiative, evidence synthesis producers could respond to requests to produce evidence syntheses through a central location, find evidence syntheses, coordinate with others, and access evidence synthesis resources.

From a demand coordination perspective, larger organizations such as the Public Health Agency were able to coordinate the questions that required evidence syntheses:

*“That evidence demand coordination was worth its weight in gold and just an extraordinary facilitator and it created a virtuous cycle in the sense that all of our teams felt like they were asking the right questions and were coming from the right people […] at least you knew it had gone through an internal process, it felt like you know questions were filtered to us in a very efficient way.”* [interviewee 16]

Despite the coordination that occurred over the pandemic, some felt siloed and not heard in the community:

*“A lot of groups were very siloed. I think what happens is, it’s a pandemic and each group does their own thing without thinking about what everybody else is doing.”* [interviewee 1]

In addition, many thought there was still a lot of duplication of efforts during the pandemic. Participants noted the biggest barrier was the difficulty finding out what evidence syntheses were already being done. Some also mentioned that timing didn’t always align so duplication occurred:

*“In spite of best efforts, there was a fair amount of wheel recreation. The notion behind COVID-END was very good but it varied a little bit in how well it worked for different things because sometimes the timelines at the COVID-END side were longer than the timelines at the user organization side and so people would still have to do something just because of the pressure system that we were in. So although the idea was the right one, there’s still some challenges in terms of trying to get everyone on the same page in terms of urgency and where the trade-offs are in terms of urgency and rigor.”* [interviewee 7]

Many expressed the importance of maintaining these networks and collaborations outside of the pandemic to not only reduce duplication, but ensure that if another emergency were to arise, the evidence synthesis community is prepared to respond.

## Discussion

This qualitative study describes how evidence synthesis practices in Canada evolved and adapted during the pandemic to produce timely evidence syntheses in a challenging environment. Evidence syntheses producers adapted their processes and products to meet the demands of different types of requests for syntheses. The results from this study are in line with two other qualitative studies looking at the production and use of evidence synthesis during the pandemic [27, 32]. This study adds additional value as the interviews were conducted later in the pandemic to encompass perspectives as the pandemic progressed, and focused on the entire country of Canada to enable more themes to potentially emerge where available. No themes that were unique to individual provinces or types or organizations were identified. The limitations of this research include a small sample size and a focus solely on evidence synthesis producers in Canada. It is unclear if the small sample size was fully representative of the diversity of experiences within this multicultural context. Future investigations aiming to inform evidence synthesis ecosystems should encompass both producers and users for a comprehensive understanding.

Although the field of evidence synthesis was able to adapt during a crisis, questions still remain as to what adaptations are acceptable, both during emergencies and more routine operations. Not unexpectedly, rapid reviews were used heavily during the pandemic to aide decision-makers needing evidence urgently [26, 33]. While there is existing guidance and standards on how to conduct a rapid review [34, 35], this was not uniformly implemented within the community, as shown in this study. In addition, there is limited evidence about which streamlined processes within a rapid review introduce lower uncertainty while still yielding a valuable product for policy-makers [36]. Also, in terms of their ability to inform policy, the extent to which rapid reviews differ in comparison to systematic reviews is not yet clear. Research studies on this topic have started to emerge over the past decade [36–41]; however, this is an area that will require a lot more research. Living reviews also gained a lot more traction during the pandemic as they are ideal when research evidence is emerging rapidly. Similar to rapid reviews, there is a lack of agreement and practice within the community on standardized methods for continuously adding studies through a living review and when it is appropriate to stop updating [42, 43]. This study showed that during the pandemic, there was a need to balance the rigor associated with systematic reviews with producing timely evidence syntheses, which led to the creation of more rapid and living reviews. The pandemic showcased that in addition to it being possible to produce evidence syntheses rapidly, there is a strong appetite for these types of products. However, as we move out of the public health emergency, it is important to determine if and what the tradeoffs are for conducting rapid reviews, which review types are appropriate under what circumstances, and moving towards standardized and accepted methods for rapid and living reviews both within the evidence synthesis community and for those requesting and using evidence syntheses. While there was no indication of an increase in the production of scoping or mapping reviews in this study, there has been an overall increase in their use over the past decade [44, 45], suggesting these types of evidence syntheses should also be considered as the evidence synthesis field evolves.

There were also many questions around the use of preprints. During the pandemic, preprints were how the newest research was disseminated. However, there was significant worry about the quality and credibility of preprints for use in decision-making as they did not go through the formal peer-review process. It is important to establish if and how preprints should be used in evidence synthesis and under what circumstances. Research is starting to emerge comparing the quality and data of preprints to their published counterparts, with current results showing they are comparable [46–48]. In addition, this study showed there were concerns about the studies being poorly conducted and reported, particularly during the beginning of pandemic which has also be documented in the literature [49, 50].

In this study, many participants expressed that there was often not time or the right tools to perform quality assessment as part of evidence syntheses. At the onset of the pandemic, definitive forms of evidence such as randomized controlled trials were lacking, making it challenging to establish certainty of evidence based on existing tools for assessing evidence credibility. Public health policy decisions relied on bodies of evidence derived from non-randomized study (NRS) designs, which are susceptible to different forms of bias than randomized designs. While tools such as ROBINS-I, the Newcastle-Ottawa Scale, and Joanna Briggs Institute appraisal tools are available to assess bias in NRS, they lack validation and are not standardized within the evidence synthesis community [51–53]. This issue is well known within the evidence synthesis community with work underway to address it [54, 55], however; this is an area that requires immediate attention as many public health policy decisions rely on evidence from NRS designs and need validated and standardized tools to assess the quality of the evidence.

Many adaptations that occurred during the pandemic were viewed as positive and participants saw value in continuing once the pandemic ended. While AI offers the potential to reduce resource use and produce evidence synthesis in less time, it is has not been widely adopted within the field of evidence synthesis [56, 57]. This is due to multiple factors such as availability, known challenges of using AI in evidence synthesis, and significant doubt on the utility of AI within the community of reviewers [56, 57]. Although there was an uptake in AI to assist with tasks such as screening which have previously proven benefits in time savings and workload reductions, there continued to be limited use in areas that have been challenging to show a benefit to using AI over humans such as data extraction and quality assessment. However, there may be other reasons as to why AI was not utilized more by these groups that were not explored in this study such as policies for using AI within the organization, confidentiality concerns, or a lack of funding to acquire the tools. The second was the use of patient-citizen partners in the evidence synthesis process. The benefits of using patient and citizen involvement in research has been well established; however, less is known about its impact and appropriateness in evidence synthesis [58, 59]. While there have been studies on the benefits and challenges to using patient and citizen partners in the evidence synthesis process, this is not uniformly practiced across the evidence synthesis community as shown in this study and is often dependent on the research question and stakeholders [58, 60]. Lastly, the collaboration and coordination within the evidence synthesis community was viewed as a tremendous adaptation during the pandemic that should continue to be developed and promoted.

### Future directions

Continuing to establish the evidence synthesis community is essential for promoting methodological rigor, fostering collaboration, building capacity, enhancing transparency, and addressing research priorities. On the back of the success of COVID-END, the time-limited network that coordinated evidence syntheses groups from around the world during the pandemic, is the creation of the Global Evidence Commission. The Commission along with other international evidence synthesis collaborations aims to improve the use of research evidence, both in routine times and in future global crises [61, 62]. These commitments to faster and more accessible evidence aim to build a new evidence ecosystem that bridges the gap between evidence synthesis communities, primary researchers, guideline developers, health technology assessment agencies, and health policy authorities [63]. Utilizing the lessons learned from this pandemic as showcased in this study and others will be imperative for the evidence synthesis field to progress further. This could include additional research comparing evidence synthesis production in different countries, additional research on the interplay between evidence synthesis producers and users, and the establishment of a community that fosters collaboration, minimizes redundancy, and generates valuable evidence syntheses for evidence-based decision-making.

## Conclusion

This qualitative study sheds light on the dynamic landscape of evidence synthesis in the context of the COVID-19 pandemic in Canada. Through an exploration of the evolution and adaptation of evidence syntheses processes and the challenges encountered, valuable insights have been gained into the resilience and adaptability of the evidence synthesis field. In addition, many areas of improvement have been identified such as the need for standardized and accepted methods for rapid and living reviews within the evidence synthesis community and the lack of validated quality assessment tools for study designs commonly used in public health research. By focusing on the perspectives of those directly involved in producing evidence syntheses, this research provides a nuanced understanding of the challenges faced and the strategies employed to ensure the continued generation of evidence syntheses for input into evidence-based decision making during a public health emergency. Moving forward, the lessons learned from this study in combination with others, can inform future strategies for improving evidence synthesis practices under the pressure of an emergency and during regular timelines.

## Supporting information

S1 Appendix Semi-structured interviews and codebook. (DOCX)

S2 Appendix COREQ (COnsolidated criteria for REporting Qualitative research) checklist. (PDF)

## References

[1] Corrin T, Ayache D, Baumeister A, Young K, Pussegoda K, Ahmad R, et al. COVID-19 literature surveillance-A framework to manage the literature and support evidence-based decision-making on a rapidly evolving public health topic. Can Commun Dis Rep. 2023 Jan 5;49(1):5–9. doi: 10.14745/ccdr.v49i01a02 36815866 PMC9902036

[2] Wang P, Tian D. Bibliometric analysis of global scientific research on COVID-19, J. Biosaf. Biosecurity. 2021 Jun;3(1):4–9. doi: 10.1016/j.jobb.2020.12.002 PMC7825845 33521590

[3] Schippers MC, Rus DC. Optimizing Decision-Making Processes in Times of COVID-19: Using Reflexivity to Counteract Information-Processing Failures. Front Psychol. 2021 Jun 22;12:650525. doi: 10.3389/fpsyg.2021.650525 34239479 PMC8258315

[4] Vickery J, Atkinson P, Lin L, Rubin O, Upshur R, Yeoh EK, et al. Challenges to evidence-informed decision-making in the context of pandemics: qualitative study of COVID-19 policy advisor perspectives. BMJ Glob Health. 2022 Apr;7(4):e008268. doi: 10.1136/bmjgh-2021-008268 35450862 PMC9023846

[5] Rubin O, Errett NA, Upshur R, Baekkeskov E. The challenges facing evidence-based decision making in the initial response to COVID-19. Scandinavian Journal of Public Health. 2021;49(7):790–796. doi: 10.1177/1403494821997227 33685289

[6] Pacheco-Barrios K, Fregni F. Evidence-based decision making during COVID-19 pandemic. Princ Pract Clin Res. 2020 Jan-Apr;6(1):1–2. doi: 10.21801/ppcrj.2020.61.1 32766450 PMC7406121

[7] Khalil H, Tamara L, Rada G, Akl EA. Challenges of evidence synthesis during the 2020 COVID pandemic: a scoping review. J Clin Epidemiol. 2022 Feb;142:10–8. doi: 10.1016/j.jclinepi.2021.10.017 34718121 PMC8550900

[8] Evidence Synthesis International. What is Evidence Synthesis? [cited 29 September 2024]. In: Evidence Synthesis International [Internet]. Available from: https://evidencesynthesis.org/what-is-evidence-synthesis/

[9] Sur RL, Dahm P. History of evidence-based medicine. Indian J Urol. 2011 Oct;27(4):487–9. doi: 10.4103/0970-1591.91438 22279315 PMC3263217

[10] Burns PB, Rohrich RJ, Chung KC. The Levels of Evidence and Their Role in Evidence-Based Medicine. Plast Reconstr Surg. 2011 Jul;128(1): 305–10. doi: 10.1097/PRS.0b013e318219c171 21701348 PMC3124652

[11] Evans D. Hierarchy of evidence: a framework for ranking evidence evaluating healthcare interventions. J Clinical Nurs. 2003 Jan;12(1):77–84. doi: 10.1046/j.1365-2702.2003.00662.x 12519253

[12] Moher D, Stewart L, Shekelle P. All in the Family: systematic reviews, rapid reviews, scoping reviews, realist reviews, and more. Syst Rev. 2015;4(1):183. doi: 10.1186/s13643-015-0163-7 26693720 PMC4688988

[13] Michelson M, Reuter K. The significant cost of systematic reviews and meta-analyses: A call for greater involvement of machine learning to assess the promise of clinical trials. Contemp. Clin. Trials Commun. 2019 Dec;16. doi: 10.1016/j.conctc.2019.100443 PMC6722281 31497675

[14] Tricco AC, Cardoso R, Thomas SM, Motiwala S, Sullivan S, Kealey MR, et al. Barriers and facilitators to uptake of systematic reviews by policy makers and health care managers: a scoping review. Implementation Sci. 2015 Jan;11(4). doi: 10.1186/s13012-016-0370-1 PMC4709874 26753923

[15] Poder TG, Rhainds M, Bellemare CA, Deblois S, Hammana I, Safianyk C, et al. Experiences of Using Cochrane Systematic Reviews by Local HTA Units. Int J Health Policy Manag. 2022;11(2):112–117. doi: 10.34172/ijhpm.2020.133 32772006 PMC9278610

[16] Gurbaxani BM, Hill AN, Patel P. Unpacking Cochrane’s Update on Masks and COVID-19. Am J Public Health. 2023 Oct;113(10):1074–8. doi: 10.2105/AJPH.2023.307377 37672741 PMC10484132

[17] Oreskes N. What Went Wrong with a Highly Publicized COVID Mask Analysis? 2023 Nov 1 [cited 05 June 2024]. In: Scientific American [Internet]. Available from: https://www.scientificamerican.com/article/what-went-wrong-with-a-highly-publicized-covid-mask-analysis/.

[18] Husson H, Howarth C, Neil-Sztramko S, Dobbins M. The National Collaborating Centre for Methods and Tools (NCCMT): Supporting evidence-informed decision-making in public health in Canada. Can Commun Dis Rep. 2021;47(5/6):292–46. doi: 10.14745/ccdr.v47i56a08 34220355 PMC8219060

[19] Bhatia D, Allin S, Di Ruggiero E. Mobilization of science advice by the Canadian federal government to support the COVID-19 pandemic response. Humanit Soc Sci Commun. 2023;10(1):19. doi: 10.1057/s41599-023-01501-8 36687774 PMC9844194

[20] Government of Canada. Overview of Canada’s COVID-19 Economic Response Plan. [cited 29 September 2024]. In: Government of Canada. Available from: https://www.canada.ca/en/department-finance/services/publications/economic-fiscal-snapshot/overview-economic-response-plan.html.

[21] MacAulay M, Fafard P, Cassola A, Palkovits M. Analysing the ‘follow the science’ rhetoric of government responses to COVID-19. Policy & Politics. 2023 Jun;51(3):466–485. doi: 10.1332/030557321X16831146677554

[22] Abbott R, Bethel A, Rogers M, Whear R, Orr N, Shaw L, et al. Characteristics, quality and volume of the first 5 months of the COVID-19 evidence synthesis infodemic: a meta-research study. BMJ Evidence-Based Medicine. 2022 Jun; 27(3):169. doi: 10.1136/bmjebm-2021-111710 34083212 PMC9132873

[23] Pearson H. How COVID broke the evidence pipeline. Nature. 2021;593:182–5. doi: 10.1038/d41586-021-01246-x 33981057

[24] Scheirer W. A pandemic of bad science. Bull At Sci. 2020 Jul;76(4):175–84. doi: 10.1080/00963402.2020.1778361

[25] Rehfuess EA, Burns JB, Pfadenhauer LM, Krishnaratne S, Littlecott H, Meerpohl JJ, et al. Lessons learnt: Undertaking rapid reviews on public health and social measures during a global pandemic. Res Synth Methods. 2022 Sep;13(5):558–72. doi: 10.1002/jrsm.1580 35704478 PMC9349463

[26] Tricco AC, Garritty CM, Boulos L, Lockwood C, Wilson M, McGowan J, et al. Rapid review methods more challenging during COVID-19: commentary with a focus on 8 knowledge synthesis steps. J Clin Epidemiol. 2020 Oct;126:177–83. doi: 10.1016/j.jclinepi.2020.06.029 32615209 PMC7836683

[27] McSween-Cadieux E, Lane J, Hong QN, Houle A-A, Lauzier-Jobin F, Saint-Pierre Mousset E, et al. Production and use of rapid responses during the COVID-19 pandemic in Quebec (Canada): perspectives from evidence synthesis producers and decision makers. Health Res Policy Sys. 2024;22(1):22. doi: 10.1186/s12961-024-01105-x 38351054 PMC10863098

[28] Hennink M, Kaiser BN. Sample sizes for saturation in qualitative research: A sysetmatic review of empirial rests. Soc. Sci. Med. 2022 Jan;292:114523. doi: 10.1016/j.socscimed.2021.114523 34785096

[29] Saunders B, Sim J, Kingstone T, Baker S, Waterfield J, Bartlam B, et al. Saturation in qualitative research: exploring its conceptualization and operalization. Qual Quant. 2018;52(4): 1893–1907. doi: 10.1007/s11135-017-0574-8 29937585 PMC5993836

[30] Gaudet S, Robert D. A journey through qualitative research. SAGE Publications Ltd; 2018. doi: 10.4135/9781529716733

[31] Tong A, Sainsbury P, Craig J. Consolidated criteria for reporting qualitative research (COREQ): a 32-item checklist for interviews and focus groups. Int J Qual Health Care. 2007;19(6):349–57. doi: 10.1093/intqhc/mzm042 17872937

[32] Clyne BA-O, Hynes L, Kirwan C, McGeehan M, Byrne P, Killilea M, et al. Perspectives on the production, and use, of rapid evidence in decision making during the COVID-19 pandemic: a qualitative study. BMJ Evid Based Med. 2023 Feb;28(1):48–57. doi: 10.1136/bmjebm-2021-111905 35772940 PMC9887371

[33] Fretheim A, Brurberg KG, Forland F. Rapid reviews for rapid decision-making during the coronavirus disease (COVID-19) pandemic, Norway, 2020. Euro Surveill. 2020 May 14;25(19): 2000687. doi: 10.2807/1560-7917.ES.2020.25.19.2000687 32431291 PMC7238744

[34] Chantelle G, Candyce H, Marialena T, Gerald G, Barbara N-S, Declan D, et al. Updated recommendations for the Cochrane rapid review methods guidance for rapid reviews of effectiveness. BMJ. 2024 Feb 6;384:e076335. doi: 10.1136/bmj-2023-076335 38320771

[35] National Collaborating Centre for Methods and Tools. Rapid Review Guidebook: Steps for conducting a rapid review. 2017. Available from: https://www.nccmt.ca/uploads/media/media/0001/01/a816af720e4d587e13da6bb307df8c907a5dff9a.pdf.

[36] Wagner G, Nussbaumer-Streit B, Greimel J, Ciapponi A, Gartlehner G. Trading certainty for speed—how much uncertainty are decisionmakers and guideline developers willing to accept when using rapid reviews: an international survey. BMC Med Res Methodol. 2017 Aug 14;17(1):121. doi: 10.1186/s12874-017-0406-5 28806999 PMC5557322

[37] Reynen E, Robson R, Ivory J, Hwee J, Straus SE, Pham B, et al. A retrospective comparison of systematic reviews with same-topic rapid reviews. J Clin Epidemiol. 2018 Apr;96:23–34. doi: 10.1016/j.jclinepi.2017.12.001 29258906

[38] Taylor-Phillips S, Geppert J, Stinton C, Freeman K, Johnson S, Fraser H, et al. Comparison of a full systematic review versus rapid review approaches to assess a newborn screening test for tyrosinemia type 1. Res Synth Methods. 2017 Dec;8(4):475–84. doi: 10.1002/jrsm.1255 28703492

[39] Tricco AC, Zarin W, Ghassemi M, Nincic V, Lillie E, Page MJ, et al. Same family, different species: methodological conduct and quality varies according to purpose for five types of knowledge synthesis. J Clin Epidemiol. 2018 Apr;96:133–42. doi: 10.1016/j.jclinepi.2017.10.014 29103958

[40] Ganann R, Ciliska D, Thomas H. Expediting systematic reviews: methods and implications of rapid reviews. Implement Sci. 2010;5(1):56. doi: 10.1186/1748-5908-5-56 20642853 PMC2914085

[41] Pham MT, Waddell L, Rajić A, Sargeant JM, Papadopoulos A, McEwen SA. Implications of applying methodological shortcuts to expedite systematic reviews: three case studies using systematic reviews from agri-food public health. Res Synth Methods. 2016 Dec;7(4):433–46. doi: 10.1002/jrsm.1215 27285733 PMC5215373

[42] Qingyong Z, Jianguo X, Ya G, Ming L, Luying C, Lu X, et al. Past, present and future of living systematic review: a bibliometrics analysis. BMJ Glob Health. 2022;7(10):e009378. doi: 10.1136/bmjgh-2022-009378 36220305 PMC9558789

[43] Simmonds M, Elliott JH, Synnot A, Turner T. Living Systematic Reviews. Methods Mol Biol. 2022;2345:121–34. doi: 10.1007/978-1-0716-1566-9_7 34550587

[44] Colquhoun HL, Levac D, O’Brien KK, Straus S, Tricco AC, Perrier L, et al. Scoping reviews: time for clarity in definition, methods, and reporting. J Clin Epidemiol. 2014 Dec;67(12):1291–4. doi: 10.1016/j.jclinepi.2014.03.013 25034198

[45] Campbell F, Tricco AC, Munn Z, Pollock D, Saran A, Sutton A, et al. Mapping reviews, scoping reviews, and evidence and gap maps (EGMs): the same but different—the “Big Picture” review family. Syst Rev. 2023 Mar;12(45). doi: 10.1186/s13643-023-02178-5 PMC10014395 36918977

[46] Brierley L, Nanni F, Polka JK, Dey G, Pálfy M, Fraser N, et al. Tracking changes between preprint posting and journal publication during a pandemic. PLoS Biol. 2022;20(2):e3001285. doi: 10.1371/journal.pbio.3001285 35104285 PMC8806067

[47] Lisa B, Rosa L, Louis L, Kellia C, Sally M, Matthew JP, et al. Cross-sectional study of preprints and final journal publications from COVID-19 studies: discrepancies in results reporting and spin in interpretation. BMJ Open. 2021 Jul 16;11(7):e051821. doi: 10.1136/bmjopen-2021-051821 34272226 PMC8288242

[48] Spungen H, Burton J, Schenkel S, Schriger DL. Completeness and Spin of medRxiv Preprint and Associated Published Abstracts of COVID-19 Randomized Clinical Trials. JAMA. 2023 Apr 18;329(15):1310–2. doi: 10.1001/jama.2023.1784 37071105 PMC10114049

[49] Zdravkovic M, Berger-Estilita J, Zdravkovic B, Berger D. Scientific quality of COVID-19 and SARS CoV-2 publications in the highest impact medical journals during the early phase of the pandemic: A case control study. PLoS One. 2020 Nov 5;15(11):e0241826. doi: 10.1371/journal.pone.0241826 33152034 PMC7643945

[50] Jung RG, Di Santo P, Clifford C, Prosperi-Porta G, Skanes S, Hung A, et al. Methodological quality of COVID-19 clinical research. Nat Commun. 2021 Feb;12(943) doi: 10.1038/s41467-021-21220-5 33574258 PMC7878793

[51] Aromataris E, Lockwood C, Porritt K, Pilla B, Jordan Z, editors. JBI Manual for Evidence Synthesis. 2024. Available from: 10.46658/JBIMES-20-01.

[52] Wells GS, Shea B, O’Connell D, Petersen J, Welch V. Losos M, et al. The Newcastle-Ottawa Scale (NOS) for assessing the quality of nonrandomised studies in meta-analyses. 2021 Available from: https://www.ohri.ca/programs/clinical_epidemiology/oxford.asp.

[53] Cochrane Methods. ROBINS-I tool. 2024 [cited 05 June 2024]. In: Cochrane Methods [Internet]. Available from: https://methods.cochrane.org/robins-i.

[54] Cuello-Garcia CA, Santesso N, Morgan RL, Verbeek J, Thayer K, Ansari MT, et al. GRADE guidance 24 optimizing the integration of randomized and non-randomized studies of interventions in evidence syntheses and health guidelines. J Clin Epidemiol. 2022 Feb;142:200–8. doi: 10.1016/j.jclinepi.2021.11.026 34800676 PMC8982640

[55] Hilton Boon M, Burns J, Craig P, Griebler U, Heise TL, Vittal Katikireddi S, et al. Value and Challenges of Using Observational Studies in Systematic Reviews of Public Health Interventions. Am J Public Health. 2022 Apr;112(4):548–52. doi: 10.2105/AJPH.2021.306658 35319925 PMC8961824

[56] Blaizot A, Veettil SK, Saidoung P, Moreno-Garcia CF, Wiratunga N, Aceves-Martins M, et al. Using artificial intelligence methods for systematic review in health sciences: A systematic review. Res Synth Methods. 2022 May;13(3):353–62. doi: 10.1002/jrsm.1553 35174972

[57] European Centre for Disease Prevention and Control. Use and impact of new technologies for evidence synthesis: Literature review and qualitative data collection. Stockholm; ECDC; 2022. Available from: https://www.ecdc.europa.eu/en/publications-data/use-and-impact-new-technologies-evidence.

[58] Agyei-Manu E, Atkins N, Lee B, Rostron J, Dozier M, Smith M, et al. The benefits, challenges, and best practice for patient and public involvement in evidence synthesis: A systematic review and thematic synthesis. Health Expect. 2023 Aug;26(4):1436–52. doi: 10.1111/hex.13787 37260191 PMC10349234

[59] Boden C, Edmonds AM, Porter T, Bath B, Dunn K, Gerrard A, et al. Patient partners’ perspectives of meaningful engagement in synthesis reviews: A patient-oriented rapid review. Health Expect. 2021 Aug;24(4):1056–71. doi: 10.1111/hex.13279 34048618 PMC8369105

[60] Cottrell E, Whitlock E, Kato E, Uhl S, Belinson S, Chang C, et al. AHRQ Methods for Effective Health Care. Defining the Benefits of Stakeholder Engagement in Systematic Reviews. Rockville (MD): Agency for Healthcare Research and Quality (US); 2014 Mar. Report No: 14-EHC0060EF. . 24783309

[61] Global Commission on Evidence to Address Societal Changes. The Evidence Commission report: A wake-up call and path forward for decisionmakers, evidence intermediaries, and impact-oriented evidence producers. Hamilton: McMaster Health Forum, 2022. Available from: https://www.mcmasterforum.org/docs/default-source/evidence-commission/evidence-commission-report.pdf?sfvrsn=2fb92517_11.

[62] Campbell Collaboration. Stepping up evidence synthesis: faster, cheaper and more useful 2023. 2023 Nov 15 [cited 05 June 2024]. Available from: https://www.campbellcollaboration.org/news-and-events/news/stepping-up-evidence-synthesis.html.

[63] Ravaud P, Créquit P, Williams HC, Meerpohl J, Craig JC, Boutron I. Future of evidence ecosystem series: 3. From an evidence synthesis ecosystem to an evidence ecosystem. J Clin Epidemiol. 2020 Jul;123:153–61. doi: 10.1016/j.jclinepi.2020.01.027 32147384

