# Supplementary material for: The evolution and adaptation of evidence synthesis during the COVID-19 pandemic in Canada: Perspectives of evidence synthesis producers
Source: PLoS One. 2024 Nov 27;19(11):e0314657. doi: 10.1371/journal.pone.0314657 (PMC11602041; doi:10.1371/journal.pone.0314657)
Supplement: S1 Appendix — (DOCX) [file pone.0314657.s001.docx]

# Appendix

## Semi-Structured Interview Guide

Section 1 – Mapping Knowledge Synthesis Groups

1. What knowledge synthesis group(s) do you work for?
2. What is your role within the knowledge synthesis group (e.g., job title)
3. How many people are in your knowledge synthesis group?
4. How is your knowledge synthesis group funded?
5. Can you share a brief timeline of when and how the knowledge synthesis group was created?

Section 2 - Evolution & Adaptation of Knowledge Synthesis

Is it safe to assume your group existed before the pandemic?

If the knowledge synthesis group existed before COVID-19 pandemic:

1. Can you outline the main goals/mandates/objectives of your knowledge synthesis group prior to the COVID-19 pandemic?
   1. Can you describe any changes to these goals/mandates/objectives as a result of the COVID-19 pandemic?
2. Can you specify the topics your group produced knowledge synthesis products on prior to the COVID-19 pandemic?
   1. During the COVID-19 pandemic, did your group work on COVID-19 topics, other topics, or a combination of the two?
   2. Can you tell me when and how your group started to work on COVID-19 topics?
   3. Please describe how your group prioritized knowledge synthesis topics.
3. Can you tell me about types of knowledge synthesis products your group produced both before and during the COVID-19 pandemic?
   1. Thinking about Open Science, can you describe your groups’ policies or procedures on open science for protocols for synthesis products before the COVID-19 pandemic?
   2. Can you identify if any of your methods or processes changed or evolved from pre-COVID-19 pandemic to COVID-19 pandemic knowledge synthesis work?
4. Can you specify any other types of knowledge synthesis work your group did prior to the COVID-19 pandemic such as developing methods or training?
   1. How this change as a result of the COVID-19 pandemic?
5. What other knowledge synthesis groups in Canada did you collaborate with before the pandemic and during the pandemic?
6. Prior to starting a new COVID-19 knowledge synthesis project, how would your group try to figure out if the topic was already underway or completed by another group?
7. Can you describe any barriers or obstacles to your knowledge synthesis work during the COVID-19 pandemic?
   1. Can you identify any resources or ideas that would have been beneficial in helping with some of these barriers or obstacles?
8. Can you describe how your group adapted during the COVID-19 pandemic?
   1. Describe any resources that were of particular assistance to your group to adapt.
9. Can you explain if and how any of the changes or adaptations made as a result of the COVID-19 pandemic can be used in your future non-COVID-19 synthesis work?
10. What is your group working on now?
11. What are the plans or hopes for your group post-COVID-19 pandemic?

If the knowledge synthesis group did not exist before COVID-19 pandemic:

1. How was your knowledge synthesis group created?
2. When was your knowledge synthesis group created?
3. Can you tell me about the main goals/mandates/objectives of your knowledge synthesis group (did this change at all throughout the pandemic)
4. Can you specify the types of knowledge synthesis products your group produces (and how this changed over the course of the pandemic)?
   1. Can you describe your groups’ policies or procedures on open science for protocols and completed synthesis products?
   2. How did your methods or processes change or evolve during the COVID-19 pandemic?
5. Can you outline any other types of knowledge synthesis work your group did such as developing methods or training?
   1. How did this change throughout the COVID-19 pandemic?
6. What other knowledge synthesis groups in Canada did you collaborate with?
   1. How did this change throughout the COVID-19 pandemic?
7. Prior to starting a new COVID-19 knowledge synthesis project, how would your group try to figure out if the topic was already underway or completed by another group?
8. Can you describe any barriers or obstacles to your knowledge synthesis work during the COVID-19 pandemic?
   1. Can you identify any resources or ideas that would have been beneficial in helping with some of these barriers or obstacles?
9. Can you describe how your group adapted during the COVID-19 pandemic?
   1. Describe any resources that were of particular assistance to your group to adapt
10. What is your group working on now?
11. What are the plans for your group post-COVID-19 pandemic?

Section 3 - Implications for Evidence-Based Decision Making

All of the questions below are specific to COVID-19 knowledge synthesis work.

1. Who were the end-users of your knowledge synthesis products or training?
2. How were your knowledge synthesis products made available to end-users?
3. Can you describe if or how your knowledge synthesis products were used in evidence-based decision-making?
4. Thinking specifically about the field of knowledge synthesis, is anything that can be done to improve knowledge synthesis practices and/or dissemination in Canada during a future pandemic or emergency situation?

Section 4 – Closing

One final question before we end the interview for today. I am going to show you a list of knowledge synthesis groups organized by province. Can you think of any groups that we are missing? A sincere thank you for your time today, it is greatly appreciated. Would it be possible to follow up at a later time if any additional questions arise or clarification is needed?

## Codebook

Table 1: Codebook for thematic analysis

| **Theme** | **Code** | **Definition** | **Notes** | **Examples (Hypothetical)** | **Examples (Real)** |
| --- | --- | --- | --- | --- | --- |
| Organization dynamics | Inception | When, how, and why the KS team was created |  | “Our team was created in 2012 to produce evidence syntheses for senior management” | “How our group came about or came to fruition is we're actually, we have connections with the X Research Institute and there was actually our knowledge synthesis group based there and then that group actually just ended up moving over to the University of X. So instead of being housed at the X Research Institute, it moved to the University of X” |
|  | Governance | The framework of the KS group | This includes structural dynamics (e.g., how the group or projects are organized), team dynamics (e.g., how many people are on the team, expertise), and institutional dynamics (e.g., where the group fits in the organization) | “Our group consists of 8 individuals, 4 are students and 4 are full-time staff” | “We are still trying to figure out what our what our governance structure is looking like, but right now we have um one group that works under X, which is a project under um John Smith and Jane Doe looking at the COVID response and network and addressing some of those key questions” |
|  | Goals/  mandates | The goals, mandates, and objectives of the KS group |  | “The purpose of our group is to be a recognized world leader in high quality evidence synthesis, including innovative methods research, to inform decision-making in healthcare” | “Our goal is to conduct high quality evidence synthesis and advancing scholarship, so I think tied to the University of X we're looking at innovation of method, advancing scholarship and making sure that we're connecting, and the syntheses are of the highest quality” |
|  | Resources | What resources were used or needed | Any assests (e.g., materials, funding, staff) to help improve the operations of the KS group | “We receive funding for our projects exclusively through government grants” | “Like where do you go for all your you know, all your resources, cause there's lots of things going on, and it's kind of having it all in one spot would be nice I think sometimes” |
| Methods/  processes | Prioritization | How and why KS work was prioritized |  | “Projects are prioritized by our senior management team. Our group is provided with a list of which projects need to be completed and by when” | “In terms of all the different types of projects that we had on the go, we normally have research associates that are assigned to those different buckets that you organize, and we prioritize based on timeline and urgency so if there's something that needs to be done at a certain time then we might have all hands-on deck trying to meet that specific deadline” |
|  | Requests | How and why KS products were requested | Specific to how and why requests were being made | “Requests for evidence syntheses are funneled through our administrative associate who collects and presents them at our weekly meetings for consideration” | “Basically, we get review requests all the time and we serve as an internal hub for that” |
|  | Open science | Policies, procedures, and actual practices regarding open science | Open science is the movement to make scientific research and its dissemination accessible to everyone | “Open science is one of our core values. Our team really tries to uphold being very open with our research and our methods” | “So, one thing that we do is we upload everything on the open science framework, and we register our protocols prior to conducting them on open science framework or Prospero” |
|  | KS methodology | The system of methods used to conduct knowledge syntheses | Changes to or training on traditional knowledge synthesis methodologies (e.g., systematic, scoping, living, and rapid reviews | “To save time, we explored the use of AI tools to rank our references so that we screened less citations” | “I would say all of our COVID products followed more of a rapid methodology rather than a tradition you know double reviewer. Maybe cutting out, so looking to cut out the things that make a systematic review very lengthy” |
| Products | Topics | The topics covered in the KS products | Types of topics covered by the KS group (e.g., cardiovascular health) | “We focus mainly on drug and health technologies” | “Preventive health care. So, screening for diseases such as prostate cancer, hypertension. Uhm, potentially inappropriate prescriptions. So, we have a whole list of different types of topics that the X are interested in” |
|  | Types | The types of KS products produced |  | “During the pandemic we moved away from traditional reviews to focus exclusively on rapid reviews and living evidence reviews” | “We had knowledge products, they wanted a PowerPoint and a kind of a 1 pager, and then a report that was structured all around the X templates that were already circulating, had already been approved and were used” |
| Evidence-based decision-making | End-users | The end-users of the KS products | Who uses the KS products | “Our products are used by local, provincial, and federal governments” | “So, end users will be health agencies. Uhm if you had like the government, the government of Canada is a good one. You also have so for training students, other researchers as well that would be end users for methods that are our learnings and our trainings that we hope to do in the future” |
|  | Availability | How KS products were made available |  | “The end product is sent directly to stakeholders through email” | “We always publish our, well we aim to publish our research in peer reviewed journals or having a final product available to our on our open science framework site and then we also disseminate our findings on Twitters occasionally as well” |
|  | Usage & uptake | How were the KS products being used or tracked for usage/uptake? |  | “I know a lot of our products on vaccines for example has been used by the World Health Organization to make policies, decisions, and recommendations” | “So, another great example for Ebola that those types of products are used by a guideline development group or forming guidelines that will eventually, you know, inform practice and whether or not they decide to you know don and doff PPE equipment in a certain order or something along the likes of one or two gloves, those types of things” |
| KS Community | Collaboration | How did KS groups collaborate and with whom? |  | “Our network is all about building the capacity within the province to support a learning health system, which would include working with our academic partners” | “I would say we have definitely made a lot more connections internationally as a result of the pandemic. So, we've been collaborating with a lot of international groups, and I think that's the big thing 'cause I mean, we've always been collaborative with other scientists and researchers in the city but in terms of doing evidence synthesis for other teams internationally, I think that's definitely new for us” |
|  | Coordination | How was coordination of work being done or mobilized? | This can include how efforts were organized or communicated in the community, and/or duplication efforts on requests, products, or topics | “It would be great if we could have a central repository where all the groups uploaded their work to avoid duplication” | “We would go to provinces or other organizations, other jurisdictions, and actually say, have they done the same thing, have they addressed the same question? Yeah, and so if they have, we've pulled, we've pulled that into our review” |
| Theoretical | Suitability of evidence | Was the type of evidence available suitable to address the request for KS? | This can include discussions about the evidence hierarchy, how some study designs are more conducive to certain research questions etc. | “At the beginning of the pandemic there were no RCTs being conducted but we had to collect any evidence that was available to provide something to our director to work with” |  |
|  | Values | How were values incorporated into KS processes or products? | Any basic or fundamental belief that guides or motivates actions pertaining to conducting KS work | “Our group never compromises on assessing the risk of bias for any review we conduct; it is always included, or we don’t do the review at all” |  |
|  | Uncertainty | How was uncertainty dealt with and managed? |  | “We did not have time to conduct GRADE, so we put a disclaimer on our review to interpret with caution” |  |
|  | Epistemic issues | The theory of knowledge pertaining to the field of KS | This can include the nature, sources, possibilities, and limitations to knowledge in the field of KS | “It was all hands-on deck. We had groups pushing out reviews who had no business conducting them in the first place” |  |

*KS = knowledge synthesis
